# Low-Cost Versatile Microfluidic Platform for Bioorthogonal Click-Mediated Nanoassembly of Hybrid Nanosystems

**DOI:** 10.3390/nano15211663

**Published:** 2025-11-01

**Authors:** Javier González-Larre, María Amor García del Cid, Diana Benita-Donadios, Ángel Vela-Cruz, Sandra Jiménez-Falcao, Alejandro Baeza

**Affiliations:** Materials and Aerospace Production Department, Superior Technic School of Aeronautics and Space Engineering, Politechnic University of Madrid, 28040 Madrid, Spain; j.glarre@upm.es (J.G.-L.); dbenita@ucm.es (D.B.-D.);

**Keywords:** microfluidics, click chemistry, nanomedicine, 3D printing, nanoassembly

## Abstract

In recent years the global market of nanomedicine has experienced incredible growth owing to the advances in the field. This translation of the technique to the biomedical industry requires the development of production methods that deliver nanomedicines with a high degree of reproducibility between batches, combined with cost and time efficiency. The use of nanoparticles in medicine usually requires their surface functionalization to improve biocompatibility in addition to providing targeting capacities and/or stimuli-responsive behavior, among other interesting skills. Microfluidic technology has revolutionized the field both in nanomedicine synthesis and in preclinical evaluation. However, microfluidic-assisted synthetic procedures commonly require high-cost methods and equipment to fabricate the microreactors. The aim of this work is to present an ultra-low-cost microfluidic platform that permits the versatile modification of nanomaterials. To prove this approach, two different model nanoparticles with different natures: soft nanoparticles (liposomes) and rigid nanoparticles (mesoporous silica) have been decorated both with small molecules and with other nanoparticles, respectively, in order to evaluate the scope of this approach. The anchoring of the covalently attached elements has been performed using click chemistry, in compliance with the principles for further transfer to the drug industry.

## 1. Introduction

The modular assembly of hybrid nanosystems that integrate functionalities such as targeted delivery, imaging, and stimuli-responsive behavior has become a key strategy in the development of advanced nanomedicines, particularly for the treatment of solid tumors [1]. The incorporation of these functionalities into nanoparticles requires their external decoration. Conventional strategies to introduce functional groups on the surface of nanomaterials usually use widely employed chemical reactions as carbodiimide chemistry, which yield the formation of strong amide bonds [2], or glutaraldehyde crosslinking that forms pH-responsive imine bonds [3], just to quote a few methods. The main liabilities of these methodologies are their lack of specificity, cross-reactivity, and the appearance of side reactions, which compromise the efficacy of the attachment process [4].

In recent years, the application of bioorthogonal reactions, defined as those that present high specificity and are unreactive toward functional groups present within the biological milieu, has provided a powerful tool to incorporate (bio)functionalities on the surface of many different types of nanosystems [5]. Among them, click chemistry reactions involve orthogonal reactions that proceed in mild conditions, with almost quantitative yield and short times [6]. The strain-promoted azide–alkyne cycloaddition (SPAAC), a copper-free click-type reaction known for its rapid kinetics and orthogonality under physiological conditions, is one of the most effective reactions to attach biomolecules on the surface of bio and nanomaterials [5]. In the case of nanomaterial decoration, the conventional batch-based approaches often fail to offer precise control over reaction time and mixing uniformity due to the slow diffusion of the nanosystems, two factors that become critical when working with ultrafast reactions such as SPAAC.

In this context, microfluidic systems provide a valuable alternative, enabling reproducible mixing, continuous flow, and tightly regulated residence times that are especially well suited to kinetic-sensitive chemistries [7]. The use of microreactors to carry out both the own fabrication and the functionalization of nanomaterials provides an excellent control of the size, morphology, and functionalization ratio through the control of key parameters as channel geometry and diameter, flow rate, and reagents/nanomaterial concentration in each channel [8]. Additionally, microfluidic-based methods to fabricate nanoparticles significantly reduce the manufacturing costs and environmental impact as a consequence of the low amount of reagents and solvents required in the process. Finally, these methods can be straightforwardly automated, yielding easy-to-tune platforms that allow the high-throughput fabrication of nanomedicines [9]. Nevertheless, the adoption of microfluidics remains limited due to high fabrication costs, specialized equipment, and highly trained personnel required in the fabrication of microreactors by different strategies as soft lithography, laser ablation, or electron/ion beam machining, among others [10]. Three-dimensional printing based on fusion deposition modeling (FDM) is an easy-to-use, low-cost, and versatile fabrication technology that has been widely applied to many different fields, such as analytical chemistry [11], biomaterials [12], and nanomaterial fabrication [13].

The use of microfluidic technology for nanoparticle synthesis offers several advantages compared to conventional batch synthesis, which often lacks control over key parameters such as particle size distribution, low encapsulation efficiency, and batch-to-batch variability [14]. These advantages include high reproducibility due to well-controlled reaction conditions; reduced solvent consumption; improved cost efficiency and minimizing environmental impact; uniform nanoparticle size; and, potentially, better control over surface chemistry and nanoparticle activity, shorter synthesis times, the possibility of automation, and enhanced mixing within the channels. Furthermore, microfluidic devices are powerful tools not only for synthesis but also for testing in vitro models or organisms-on-a-chip [9,14,15].

Although there are several techniques for fabricating microfluidic devices, such as photolithography, microcutting, or injection molding, FDM allows rapid fabrication of microreactors with versatile geometries that can be customized to meet specific needs, all at a significantly lower cost [9]. As an example, a PLA filament and a low-cost 3D printer were used in the research presented here, resulting in considerable cost reduction. However, this approach imposes limitations on channel resolution and minimum dimensions, solvent compatibility, and the risk of channel clogging [9,14,15].

**Figure 1:**
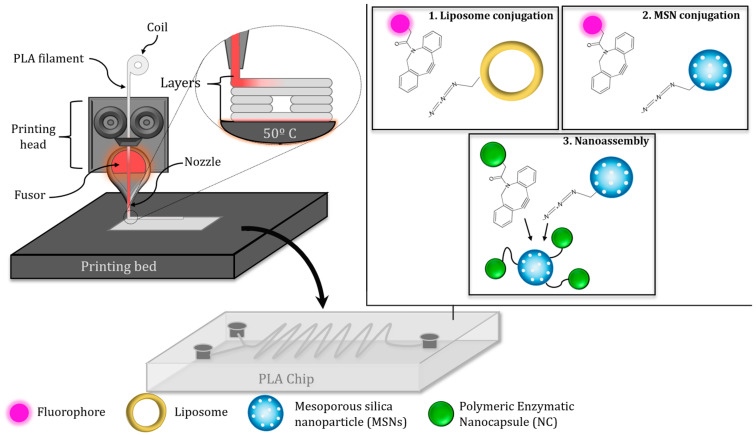
Schematic workflow of the fabrication and application of a PLA microreactor for the assembly of nanomaterials. The reactor was fabricated via FDM 3D printing using a PLA filament. The design incorporates a serpentine channel to promote mixing. Two different nanoformulations were functionalized with DBCO-modified fluorophores: liposomes and mesoporous silica nanoparticles (MSNs). The latter were also conjugated with enzyme-loaded nanocapsules (NCs) functionalized with DBCO via SPAAC inside the chip to generate multicomponent hybrid nanosystems.

In this work a novel methodology for the surface decoration of different nanomaterials using a low-cost FDM-fabricated microfluidic device is described. The microreactor presents two channels with a Y-shaped junction followed by a sinusoidal reaction chamber (Figure 1). The channels present an average section of 400–500 μm and a slightly rough surface, which is suitable to act as a micromixer that enhances the mixing process and, therefore, accelerates the reaction time. The scope of this method was evaluated employing two types of nanoparticles, which exhibited completely different natures: soft organic nanoparticles (liposomes and polymeric nanocapsules) and hard inorganic nanoparticles (mesoporous silica). Thus, the external surface of these nanoparticles was decorated with complementary clickable groups like azide and strained alkynes, depending on each case. Initially, the methodology was tested using liposomes as a soft nanoparticle model and mesoporous silica nanoparticles (MSN) as a hard-type nanoparticle model, which were previously decorated with azide groups. Both types of nanoparticles were efficiently decorated in short periods of time (less than 5 min) with fluorophores labeled with dibenzocyclooctine (DBCO) as a strained alkyne, confirming the suitability of this strategy to attach organic moieties on the external surface. Then, we explore the feasibility of preparing clusters of nanoparticles within the microreactor in order to evaluate the scope of the process in a more complex situation in which both reactive species are nanoparticles that present low diffusion rates. In this case, a suspension of mesoporous silica nanoparticles decorated with azide groups was pumped through one channel, whereas DBCO-functionalized polymeric nanocapsules loaded with catalase were pumped through the second channel. The results confirmed the efficient and rapid formation of nanoassemblies composed of mesoporous silica nanoparticles coated with polymeric nanocapsules, which retain the enzymatic capacity of the housed catalase. This novel strategy provides an ultra-low-cost method to functionalize and produce clusters of nanoparticles in a rapid and controlled manner.

The versatility of the described platform extends beyond the proof-of-concept examples presented in this work. Owing to its compatibility with both soft organic nanostructures (such as liposomes or polymeric nanocapsules) and hard inorganic nanoparticles (such as mesoporous silica), the system could be readily adapted to the synthesis and surface functionalization of different nanosystems, including metallic nanoparticles [16] and polymeric micelles [17]. This approach could be used for applications in biosensing, disease diagnosis, tissue and organ modeling, and even in toxicity studies of biomedical nanomaterials [18,19,20]. In this context, these tools fulfil both requirements, as versatile reactors for nanoparticle synthesis and as physiologically relevant experimental models, which highlights their potential as universal platforms bridging nanomedicine, diagnostics, and organ modeling.

## 2. Materials and Methods

### 2.1. Reagents

The following reagents were used in this study: 1,2-distearoyl-sn-glycero-3-phosphocholine (DSPC); 1,2-distearoyl-sn-glycero-3-phosphoethanolamine-N-[azide(polyethylene glycol)-2000] (DSPE-PEG(2000)-N_3_); polycarbonate membranes 0.1 μm, 19 mm; purchased from Avanti Polar Lipids, Alabaster, AL, USA; and quick-dry epoxy resin (Araldite^®^, Ceys^®^, Alcobendas, Madrid), 0.5 × 16 mm stainless steel needles (Agani™, Terumo^®^, Tokyo, Japan). The rest of the reagents were purchased from Sigma-Aldrich, St. Louis, MO, USA: cholesterol, 5-carboxytetramethylrhodamine-dibenzocyclooctyne (DBCO-TAMRA), chloroform, tetraethyl orthosilicate (TEOS), 3-aminopropyltriethoxysilane (APTES), cetyltrimethylammonium bromide (CTAB), ammonium hydroxide (NH_4_OH, 0.32 M), ammonium nitrate (NH_4_NO_3_), ethanol, 6-azidohexanoic acid (6-AHA), N,N′-diisopropylcarbodiimide (DIC), N-hydroxysuccinimide (NHS), N,N-dimethylformamide (DMF), catalase from bovine liver (Cat), acrylic acid N-hydroxysuccinimide ester (NHS-acrylic acid), acrylamide (AA), aminoethyl methacrylamide (AM), N,N′-methylenebisacrylamide (MBA), N,N,N′,N′-tetramethylethylenediamine (TMEDA), ammonium persulfate (APS), dibenzocyclooctyne-N-hydroxysuccinimide ester (DBCO-NHS), phosphate-buffered saline (PBS), dimethyl sulfoxide (DMSO), hydrogen peroxide (H_2_O_2_), Sephadex G-25, Amicon^®^ Ultra centrifugal filters (MWCO 30 kDa), nitrogen gas (N_2_), and polylactic acid filament coil (PLA).

### 2.2. Nanoparticle Synthesis

#### 2.2.1. Synthesis of Azide-Functionalized Small Unilamellar Liposomes (Lip-N_3_)

After weighing the lipids used for the preparation of the liposomes (4 mg of DSPC, 3.3 mg of DSPE-N_3_, and 0.6 mg of cholesterol), the lipid mixture was dissolved in 2 mL of chloroform as the organic phase in a 100 mL round-bottom flask. The solvent was evaporated using a rotary evaporator, forming a thin lipid film on the inner wall of the flask. Then, 2 mL of filtered PBS was added as the aqueous phase, and the dispersion was sonicated using a probe sonicator at 40% amplitude with 0.8 s pulses for 20 min. Due to the moderately high polydispersity of the resulting suspension, the liposome batch was extruded through a 100 nm polycarbonate membrane to obtain a more homogeneous size distribution of azide liposomes (**Lip-N_3_**).

The extruded suspension was then characterized using a dynamic light scattering (DLS) and electrophoretic light scattering (ELS) BeNano 90 Zeta equipment (BetterSize, Costa Mesa, CA, USA) to determine the hydrodynamic diameter and ζ-potential, respectively.

#### 2.2.2. Synthesis of Azide-Functionalized Mesoporous Silica Nanoparticles (MSN-N_3_)

Mesoporous silica nanoparticle type MCM-41 was synthesized via a modified Stöber method. Briefly, 0.29 g of CTAB was dissolved in 150 mL of distilled water containing 0.32 M NH_4_OH under vigorous stirring at 50 °C in a tall, 250 mL glass beaker, sealed with Parafilm to prevent evaporation. After complete dissolution, Parafilm was removed, and 3 mL of an ethanolic mixture containing 950 µL of TEOS and 50 µL of APTES was added dropwise. The reaction was maintained under continuous stirring at room temperature for 2 h at 50 °C, and then statically for 24 h. Subsequently, the dispersion was transferred to a sealed glass bottle and incubated statically at 50 °C in a stove for an additional 24 h to promote structural reorganization and improve mesopore ordering. The resulting nanoparticles were recovered by centrifugation and washed three times with 96% ethanol.

Surfactant extraction was performed by ionic exchange using 50 mL of ammonium nitrate in 96% ethanol (20 g/L). The extraction was carried out in three successive steps: a short 2 h reflux, followed by a 24 h reflux, and a final 2 h reflux incubation, all at 60 °C. After each extraction step, the obtained amino-functionalized silica nanoparticles (**MSN-NH_2_**) were centrifuged and resuspended in fresh ammonium nitrate solution in order to continue with the subsequent extraction, and finally in absolute ethanol to store the obtained product at 4 °C.

For azide surface modification, 120 mg of the extracted aminated particles were redispersed in 2 mL of DMF and reacted with 27.5 µL of preactivated 6-AHA (previously incubated for 45 min at room temperature in the presence of 72.6 µL of DIC and 54 mg of NHS), at room temperature for 18 h (**MSN-N_3_**). Following the reaction, the MSN-N3 were washed three times with ethanol to remove unreacted reagents and stored as a stable dispersion in absolute ethanol at 4 °C. The final nanoparticle concentration was determined by drying a known volume of the suspension in a stove at 70 °C and weighing the dry residue.

Both amino- and azide-functionalized nanoparticles were characterized by dynamic light scattering (DLS) and ζ potential analysis.

#### 2.2.3. Synthesis of Polymeric, DBCO-Functionalized Catalase Nanocapsules (DBCO-CatNCs)

In order to introduce acroyl groups on the surface of the enzyme, 5 mg of lyophilized catalase were dissolved in 1 mL of sodium carbonate buffer (pH 8.5). Then, 10 µL of a 6.3 mg/mL NHS-activated acrylic acid solution in DMSO was added, and the mixture was incubated under orbital shaking at room temperature for 1 h.

Following incubation, the acrylated enzyme was washed twice with carbonate buffer using Amicon^®^ ultrafiltration units (MWCO 30 kDa) by centrifugation at 2000× *g* at room temperature for 20 min per wash to remove unreacted acrylic acid. The retentate was resuspended to 1 mL in carbonate buffer.

Separately, the monomer mixture for nanocapsule formation was prepared by weighing 8.5 mg of AA, 14.9 mg of AM, and 1.54 mg of MBA. These were placed in sealed glass vials with silicone septa and subjected to vacuum/nitrogen cycles to ensure an inert atmosphere. The radical initiators, 3 µL of TMEDA and 2.28 mg of APS, were handled similarly under short vacuum/nitrogen cycles to avoid TMEDA evaporation.

Each component (monomers and initiators) was dissolved in 2 mL of degassed carbonate buffer using nitrogen-purged syringes. The catalase solution was also degassed by continuous nitrogen bubbling for 10 min. The monomer solution was then added to the catalase under orbital shaking. Finally, the initiator solution was added dropwise over 10 min while stirring continued, allowing controlled polymerization and minimizing large aggregate formation. The polymerization was allowed to proceed for 1.5 h at room temperature under inert conditions.

Afterward, the septa were opened to introduce oxygen and terminate the polymerization. The resulting dispersion was filtered through a small cotton plug using a glass Pasteur pipette to remove macroscopic polymer aggregates. The nanocapsules were further purified via three cycles of centrifugation and washing with PBS using Amicon^®^ filters (MWCO 30 kDa). The final product (**CatNCs**)was resuspended to 1 mL in PBS and characterized by DLS and ELS, then stored at 4 °C for further use.

To introduce strained alkynes for SPAAC conjugation, CatNCs in 2 mL of PBS were incubated overnight by adding 10 µL of a solution containing 0.1 mg of DBCO-NHS ester dissolved in 100 µL of DMSO under orbital agitation at room temperature, yielding **DBCO-CatNCs**. After incubation, the nanocapsules were washed again with PBS using Amicon^®^ filtration, resuspended to 1 mL in PBS, characterized by DLS, and stored refrigerated at 4 °C until the microfluidic assembly experiment was performed 24 h later.

### 2.3. Microreactor Fabrication

**Figure 2:**
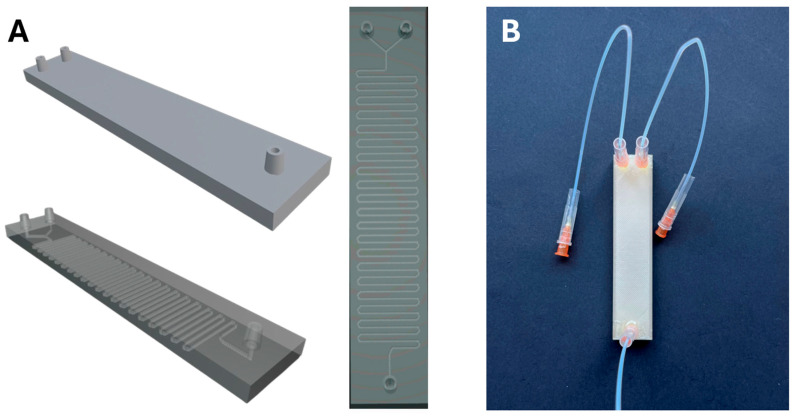
(**A**) Blender 3D models of the microreactor, including the complete model, a view of the internal channel, with integrated inlets and outlets, and a detailed top-view of the internal serpentine channel design used to promote mixing. (**B**) Photograph of a fully assembled PLA-printed microreactor with epoxy-fixed stainless steel needles and Teflon tubing.

The microfluidic device was fabricated using fused deposition modeling (FDM) technology with an Ender 3 V2 Neo^®^ (Shenzhen, China) 3D printer. Three-dimensional models were created using Blender (v4.4.1) modeling software and processed with the UltiMaker Cura software (v5.7.1). Printing parameters were as follows: Extruder nozzle width: 0.1 mm, layer height: 0.12 mm, printing bed T° = 50 °C, nozzle T° = 205 °C. Three-dimensional models and a fully printed and assembled microreactor can be seen in Figure 2.

After the microreactor was fully printed, Agani™ 0.5 × 16 mm stainless steel needles were fit into the inlets, and each needle was inserted into a Metrohm^®^ (Herisau, Switzerland) 0.5 mm caliber Teflon capillary tube in order to link the syringes from the pump to the microreactor. The caps from the needles were cut open to facilitate safe handling. Both the inlets and the capillary tubes were fixed and sealed to the needles with Araldite^®^ quick-dry epoxy resin and were allowed to dry overnight.

### 2.4. Microfluidic Functionalization of Nanomaterials with Organic Molecules

#### 2.4.1. Functionalization of Lip-N_3_ with DBCO-TAMRA [(Lip-N_3_)-(DBCO-TAMRA)]

A 5 mM solution of DBCO-TAMRA fluorophore and a 0.7 mM solution of Lip-N_3_ were prepared in filtered PBS. The liposome concentration was estimated based on the total molar amount of lipids used during vesicle synthesis. Each solution (5 mL) was loaded into separate syringes and introduced into the luer of a PLA microreactor. The flow rate was set to 0.1 mL/min to avoid overpressure within the system. The internal microchannel of the microreactor features a total of 40 turns to enhance in-line mixing of both solutions.

The output from the microreactor **(Lip-N_3_)-(DBCO-TAMRA)** was collected in an amber-protected glass vial to prevent photodegradation of the fluorophore after approximately 4.5 min. Unreacted DBCO-TAMRA was removed by size exclusion chromatography using a column packed with Sephadex G-25 and filtered PBS as the mobile phase. Seven fractions were collected until no visible color was observed in the eluate. Each fraction was analyzed by dynamic light scattering (DLS) to determine the presence of liposomes based on scattering intensity.

#### 2.4.2. Functionalization of MSN-N_3_ with DBCO-TAMRA [(MSN-N_3_)-(DBCO-TAMRA)]

The reaction between azide-functionalized mesoporous silica nanoparticles (MSN-N_3_) and DBCO-TAMRA was performed as follows: 5 mg of MSN-N_3_ were washed with filtered PBS to replace the ethanol from the stock suspension and resuspended in 2 mL of PBS immediately before the reaction to minimize silica hydrolysis. In parallel, a 5 µg/mL solution of DBCO-TAMRA was prepared in 2 mL of PBS. Both suspensions were loaded into separate syringes and co-injected into the microfluidic device at a controlled flow rate of 0.1 mL/min.

The output volume collected from the microreactor, which contained the modified liposomes **(MSN-N_3_)-(DBCO-TAMRA),** was approximately 3.9 mL and was stored in light-protected glass vials to prevent photodegradation of the fluorophore.

After the reaction, the suspension was centrifuged at 11,100× *g* for 10 min at 15 °C. The absorbance of the supernatant was measured at 545 nm using UV/Vis spectrophotometry to determine the amount of unreacted fluorophore, which was compared to the initial fluorophore input to calculate reaction efficiency. DLS and zeta potential measurements were also performed to characterize the size and surface charge of the functionalized particles. After measuring the absorbance, [(MSN-N_3_)-(DBCO-TAMRA)] was redispersed in fresh PBS buffer and incubated at room temperature with rotary agitation for 24 h to wash non-covalently bound fluorophore, and then centrifuged again under the same conditions to measure the absorbance of the supernatant again. The recorded signal was included in the final yield calculations.

In order to determine the efficiency of the microfluidic device, a comparative analysis between a conventional passive diffusion reaction between MSN-N_3_ and DBCO-TAMRA in an 8 mL glass vial and the microreactor was conducted. Each reagent was added in the same concentration and volume as in the microfluidic reactor and was allowed to react for the same time (4.5 min) without stirring at room temperature. Immediately after, the volume was transferred to a 2 mL tube and centrifuged at 11,000× *g* for 10 min at 15 °C, and the supernatant was collected to measure the functionalization yield.

### 2.5. Assembly of Hybrid Nanosystems [(MSN-N_3_)-(DBCO-CatNC)]

To generate the hybrid nanoassemblies, 0.5 mg of azide-functionalized mesoporous silica nanoparticles (MSN-N_3_) were aliquoted from the stock batch and washed several times with filtered PBS immediately before the experiment to avoid premature hydrolysis. The washed silica was resuspended in 1 mL of PBS. In parallel, 1 mg of DBCO-functionalized catalase nanocapsules (DBCO-CatNCs) were resuspended in 1 mL of PBS. Each suspension was loaded into separate syringes and simultaneously injected into the PLA microreactor through independent inlets at a flow rate of 0.1 mL/min per channel. A 2:1 ratio of capsules to silica was selected to minimize undesired crosslinking between MSN-N_3_ particles, which could lead to aggregation and sedimentation within the channel.

The product eluted from the microreactor was collected in a microcentrifuge tube and centrifuged at 11,100× *g* at 15 °C for 10 min to separate the functionalized silica from unbound nanocapsules. The resulting pellet, enriched in **[(MSN-N_3_)-(DBCO-CatNCs)**], was resuspended in 1 mL of PBS and immediately used for enzymatic activity assays.

#### 2.5.1. Enzymatic Activity Assay

To verify the successful incorporation of DBCO-CatNCs onto MSN-N_3_, a TEM characterization and an enzymatic activity assay were performed. The enzyme activity was assessed by measuring the depletion of H_2_O_2_ (28.65 mM in PBS) as a readout, monitored by UV/Vis spectrophotometry at 240 nm. The absorbance was recorded every 30 s over a 6 min interval, using 2 mL of substrate and 25 µL of enzymatic sample in a quartz cuvette, and resuspending three times prior to initiating the measurement. PBS was used as a negative control, and native free catalase as the positive control.

Samples from the microfluidic reaction composed of silica nanoparticles decorated with DBCO-CatNCs were centrifuged at 11,100× *g* for 10 min at 15 °C. The resulting pellets were resuspended in 1 mL of PBS and analyzed in order to evaluate the enzyme activity as the rate of H_2_O_2_ consumed as previously described.

#### 2.5.2. Colloidal Stability Assay

Given the known instability of MSN suspensions [21], one of the main concerns in this study was the possibility that aggregation and subsequent sedimentation may occur within the channels of the microreactor, hence the necessity to perform an analysis of the colloidal stability of the nanoassembly, in order to minimize the loss of material during the period of time in which the reaction takes place. Briefly, the collected nanoassemblies were redispersed in 2 mL of filtered PBS and allowed to sit undisturbed for ten minutes in a 2 mL Eppendorf tube, which is more than double the residence time of the reaction in the microreactor. Samples of the top layer of the supernatant were collected at established time marks and analyzed by DLS at a fixed attenuation range in order to detect scattering intensity fluctuations.

## 3. Results and Discussion

### 3.1. Synthesis of Azide-Functionalized Small Unilamellar Liposomes (Lip-N_3_) and Azide Modified Mesoporous Silica Nanoparticles (MSN-N_3_)

The lipid formulation used for the synthesis of small unilamellar liposomes included two phospholipids as structural components (DSPC and DSPE-N_3_) and cholesterol to modulate bilayer fluidity. The presence of DSPE-N_3_ enabled bioorthogonal conjugation with a complementary DBCO-functionalized fluorophore (5-TAMRA) used as a model organic molecule and for subsequent detection via UV-Vis spectroscopy. The molar ratio used was 65:20:15 for DSPC:Chol:DSPE-N_3_, respectively. Since the application of the microfluidic platform envisioned here aims to provide a low-cost, versatile platform for the modification of pharmaceutically relevant nanosystems, the DSPE-N_3_ lipid used contained a PEG-2000 spacer. The presence of PEG moieties contributes to colloidal stability and may help to reduce immune recognition by limiting opsonin adsorption, which is one of the challenges that nanoparticles face when dealing with biomedical purposes [22,23]. The liposomes obtained presented a mean hydrodynamic diameter of 82 nm (determined by DLS analysis), making them compatible with the extravasation through the fenestrations of the aberrant blood vessels surrounding solid tumors, and thus appropriate for nanomedicine use [24]. Regarding the surface charge of Lip-N_3_, a near-zero potential has been determined, −5 mV, the mean value, which can be explained by the partial blockout of the anionic phospholipids by the presence of PEG moieties [25].

Regarding the synthesis of the inorganic rigid nanoparticle used in this study as a model, mesoporous silica nanoparticles MCM-41 were synthesized via a modified Stöber method using NH_4_OH as a catalyst to promote controlled hydrolysis and condensation [26]. Two different alcoxysilanes were used as silica source: TEOS (as major component) and APTES, which provided free -NH_2_ groups that permitted further functionalization of the silica surface with the azide click group present in the 6-azidohexanoic acid molecule. Prior to the grafting of 6-azidohexanoic acid to the surface of the silica, the activation of the acid group was performed in the presence of DIC and NHS for 45 min. After this time, the active carboxylic acid group reacted with the amine group present in the surface of the silica, generating a covalent ester bond. As confirmed by the hydrodynamic diameter results, the transformation of MSN-NH_2_ to MSN-N_3_ did not significantly affect the size of the nanoparticles. However, the successful introduction of the azide group was confirmed by a shift in surface charge from 18 mV (MSN-NH_2_) to −18 mV (MSN-N_3_). These results can be observed in Table 1.

### 3.2. Microfluidic Device Characterization

**Figure 3:**
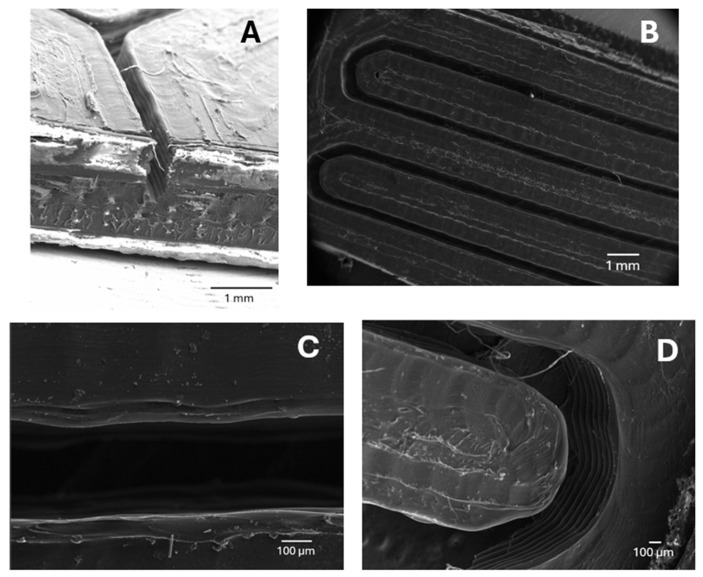
Scanning electron microscopy (SEM) images of the 3D-printed microreactor fabricated using FDM of PLA. (**A**) Cross-sectional view of a straight channel, showing the internal cavity and the multilayered filament deposition. (**B**) Top view of the meandering channel layout. (**C**) Higher magnification view of a straight section of the channel, highlighting the internal width and wall definition. (**D**) Detail of a curved region of the channel, where filament layering and surface defects are more apparent.

The geometry and resolution of the 3D-printed microfluidic microreactor were evaluated by scanning electron microscopy (SEM), as can be seen in Figure 3. The results confirmed that the fusion deposition modeling (FDM) process using PLA allows the construction of well-defined microchannels with characteristic dimensions in the range of several hundred micrometers in width. This variance can be explained by the limited resolution of the printing device, which likely introduces slight deviations in the dimensions of the printed geometries as a result of the conversion of 3D files into a stratified file compatible with the 3D printer. All images show the characteristic layered structure of the FDM process, as well as a few typical printing artifacts such as residual filaments and surface irregularities. These observations confirm that the printing resolution is enough to provide structures in the range of hundreds of micrometers, but also reveal limitations in precision and consistency, particularly in curved or fine feature regions. However, it should be noted that the microfluidic reactor presented here has been printed with a low-cost 3D printing device, and as will be explained in the subsequent discussion, these irregularities did not compromise the surface functionalization of nanoparticles.

### 3.3. Microfluidic Functionalization of Nanomaterials with Organic Molecules

**Figure 4:**
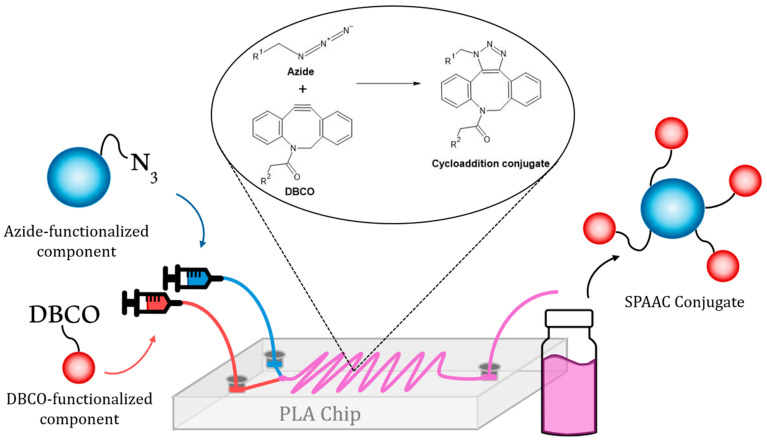
Schematic representation of the microfluidic-assisted SPAAC reaction between azide-functionalized component (blue) and DBCO-functionalized component (red). Both reactants are introduced through independent syringe pumps into the PLA-printed microreactor, where the mixing channel facilitates their conjugation via SPAAC. The resulting hybrid nanoassemblies are collected at the outlet for further use.

The strategy envisioned here consists of the surface functionalization of nanomaterials with different entities (both organic molecules and other nanoparticles) within a low-cost microfluidic reactor. Therefore, the two elements to be attached must be introduced separately by a syringe pump in order to promote the reproducibility of the injection within the reaction chamber (Figure 4). Afterward, the two elements (each one bearing a click reactive group, either an azide or an alkyne group) will meet within the chamber, and the subsequent cycloaddition reaction will occur, yielding a covalent bond responsible for the attachment of the nanoparticle and the other counterpart. Finally, the resulting modified nanomaterial will be eluted and recovered.

#### 3.3.1. Functionalization of Lip-N_3_ with DBCO-TAMRA [(Lip-N_3_)-(DBCO-TAMRA)]

Liposomes were chosen as a model of soft organic nanoparticle to be functionalized with an organic molecule because of their wide use for biomedical and pharmaceutical purposes [27,28,29], and because of their similarity to the surface of animal cells [30]. In this case, azide-modified liposomes were introduced into one inlet, and DBCO-TAMRA was introduced into the other inlet of the microreactor. TAMRA is a commonly used fluorophore for biotechnological purposes, which has a strong absorption signal at 545 nm and will be used as both the model organic molecule to be attached and the optical probe to assess the strategy proposed here [31,32,33]. This TAMRA moiety presents an alkyne group, which is going to react with the azide group present on the surface of the liposomes, yielding (Lip-N_3_)-(DBCO-TAMRA).

After the modification of Lip-N_3_ with DBCO-TAMRA, the product of the reaction [(Lip-N_3_)-(DBCO-TAMRA)] was analyzed by DLS in order to characterize the hydrodynamic diameter of the product, which did not significantly change as expected [82 nm as mean value in the case of Lip-N_3_ and 70 nm in the case of (Lip-N_3_)-(DBCO-TAMRA)]. The surface charge of (Lip-N_3_)-(DBCO-TAMRA) revealed a decrease in the potential from −5 mV in the case of Lip-N_3_ to −16 mV for the product recovered after the reaction occurred in the microfluidic platform. This shift could be attributed to the deprotonated carboxyl groups provided by the TAMRA moiety [34], confirming the modification of the liposome’s surface. The extent to which the chemical reaction had occurred was determined by the difference in the initial concentration of DBCO-TAMRA and the unbound DBCO-TAMRA eluted from the microfluidic device. The recovery of liposomes was achieved by the analysis of different fractions eluted after SEC and analyzed by DLS, confirming the presence of liposomes by the quantified kilocounts. The pooled fractions were subsequently analyzed by UV–Vis spectroscopy at 545 nm to quantify the amount of conjugated fluorophore. A calibration curve for DBCO-TAMRA was used to determine the unbound fluorophore’s concentration. Its absorbance value corresponded to 0.00441 µmol of fluorophore. Given that 0.026 µmol of DBCO-TAMRA had been introduced into the microreactor for the modification of Lip-N_3_, the amount of fluorophore quantified in the supernatant meant that 0.022 µmol of DBCO-TAMRA had been attached to Lip-N_3_. Therefore, the overall conjugation yield was approximately 85%, estimated as the rate of DBCO-TAMRA introduced in the microfluidic reactor and DBCO-TAMRA grafted to the surface of Lip-N_3_, as shown in Equation (1):

(1) $$Functionalization\;Yield=\;\frac{µmoles\;TAMRA\;attached}{µmoles\;TAMRA\;total}\times 100=\;\frac{0.022}{0.026}\times 100=85\%\;$$

Considering that a portion of the azide groups is likely located on the inner surface of the liposomal bilayer and is, therefore, inaccessible to the fluorophore under these conditions, the achieved functionalization yield implies a successful result. Note that the functionalization yield shown in Equation (1) presents no variance. This is attributed to the stoichiometrically fixation of the number of available azide groups in the liposome formulation and the nearly quantitative efficiency of the reaction with DBCO-TAMRA.

#### 3.3.2. Functionalization of MSN-N_3_ with DBCO-TAMRA-[(MSN-N_3_)-(DBCO-TAMRA)]

Besides liposomes, mesoporous silica nanoparticles were used as a model in order to test the performance of the microreactor with a “rigid” inorganic nanomaterial, because of their relevance for biotechnological applications. In the case of silica, avoiding aggregation is critical to ensure safety when nanoparticles are transported through blood capillaries or alveoli. In addition, a model with less diffusion velocity and a higher tendency to sediment, like silica, is considered worthy to be experimented with in order to establish a versatile microreactor platform [35,36,37].

Similarly to the previous experiment, the modification of the model inorganic “hard” particle was carried out using DBCO-TAMRA. Following the methodology described above, a suspension of 5 mg of silica nanoparticles was introduced into one inlet, whereas a solution of 0.016 µmol of DBCO-TAMRA was introduced into the other one. After approximately 4.5 min, the first aliquot eluted from the microreactor. Afterward, the whole sample was collected, centrifuged to recover the nanoparticles present, and the supernatant was analyzed to quantify the unbound DBCO-TAMRA. UV/VIS analysis of the supernatant revealed that 84% of the DBCO-TAMRA fluorophore successfully reacted with the complementary MSN-N_3_ (Equation (2)), indicating efficient surface coupling under mild microfluidic conditions in a short time. These results (obtained by three technical replicates) support the effectiveness of the microreactor design in promoting rapid click-conjugation.

(2) $$Functionalization\;Yield=\frac{µmoles\;TAMRA\;attached}{µmoles\;TAMRA\;total}\times 100=\frac{0.0141}{0.0167}\times 100=84\%\;\pm 15$$

To confirm the effect that the surface modification had on the surface charge of the silica nanoparticle, ζ potential measurements were performed. A substantial decrease in surface potential upon fluorophore conjugation was observed [from −18 mV in the case of MSN-N_3_ to −7 mV in the case of (MSN-N_3_)-(DBCO-TAMRA)], reflecting the neutral character of the DBCO group and the successful surface functionalization. This reduction in ζ potential likely compromised the colloidal stability of the MSNs, promoting partial aggregation and sedimentation inside the microfluidic channel. Although this might seem an undesirable outcome, in this case, it confirmed that MSN-N_3_ has been correctly modified since its surface properties had changed and therefore its behavior within the microfluidic chamber.

#### 3.3.3. Comparative Evaluation of Microreactor Efficiency

To assess the efficiency of the microfluidic platform in the synthesis of nanoassemblies, a comparative study was performed between a conventional passive diffusion reaction (Control) and the microreactor-enabled process (μF). Both conditions produced comparable functionalization yields of [(MSN-N_3_)-(DBCO-TAMRA)], with no statistically significant differences between the groups (*p* = 0.1; Mann–Whitney U Test, n = 3 for each group).

Although the difference was not significant, this outcome reinforces the suitability of the microreactor as a reliable low-cost alternative for the preparation of nanoassemblies. The fact that functionalization efficiency is not reduced compared to the conventional reaction highlights its reproducibility and robustness. Beyond equivalence in yield, the microreactor offers additional advantages intrinsic to microfluidic systems, including the possibility of modular integration of sequential reactions to incorporate different nanomaterials, thereby conferring new functionalities to the assemblies. Moreover, coupling this module in series with purification chips could streamline the process, reducing both time and reagent consumption.

### 3.4. Assembly of Hybrid Nanosystems

Despite the interesting features that nanomaterials bring, the use of a single type of nanoparticle inherently presents limitations since it excludes additional features that other nanomaterials could provide. In order to solve this issue, the development of nanotechnology has brought an imaginative approach consisting of the preparation of hybrid nanosystems bearing synergistic effects promoted by the combination of individual elements. The possibilities that arise from this perspective are unlimited, since plenty of combinations can be explored using both organic and inorganic nanomaterials [38]. This approach is of special interest in the case of functional elements that respond to stimuli or that trigger a certain response. Enzymes are a good example of functional items with special relevance in the biomedical field since a variety of enzyme-based therapies have emerged to treat various diseases in recent years. Nevertheless, the use of these biomolecules is affected by their sensitivity to degradation or loss of activity, which can be avoided by their encapsulation within a protective matrix [39]. In this work, non-degradable catalase-loaded polymeric nanocapsules (CatNCs) have been chosen as a model of “soft” active nanoparticle in order to prove the feasibility of the nanoreactor envisioned here to prepare challenging nanohybrids using SPAAC-based functionalization strategies.

**Figure 5:**
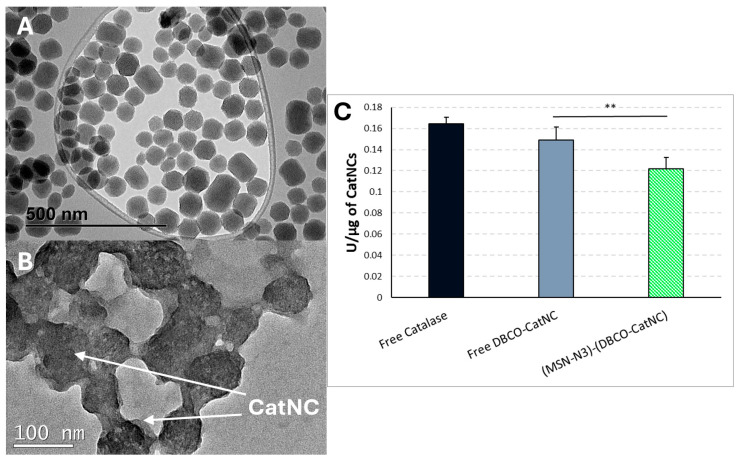
TEM images of (**A**) bare MSN-N_3_ nanoparticles, and (**B**) [MSN-N_3_)-(DBCO-CatNCs)] nanoassembly, using phosphotungstic acid as a contrast agent. (**C**) Catalase activity of different samples normalized per microgram of material (U/µg). “Free Catalase” corresponds to the unmodified native enzyme, while “Free DBCO-CatNC” represents the polymeric nanocapsules after DBCO modification. “(MSN-N_3_)-(DBCO-CatNC)” corresponds to silica nanoparticles functionalized with DBCO-CatNCs using the microfluidic chip. (n = 3 for each sample; ** *p* < 0.001, unpaired Student’s *t*-test).

The first step of the synthesis of the polymeric nanocapsules consisted of the attachment of acroyl groups on the catalase surface to provide anchoring groups for the non-degradable polymeric capsule to be synthesized above. This polymeric shell maintained the catalytic activity of the housed enzyme, preserving its action even in the presence of extreme conditions as a proteolytic environment or high temperatures [40]. As can be observed in Figure 5B, the modification of MSN-N_3_ with DBCO-CatNCs yields a texturized silica surface with uniformly distributed globular organic structures, which are the non-degradable polymeric DBCO-CatNCs. The successful assembly of the hybrid nanosystem composed of a silica core surrounded by DBCO-CatNCs was confirmed by DLS experiments, since an important growth in the hydrodynamic diameter was observed after the modification of silica nanoparticles with DBCO-CatNCs (136 nm to 267 nm, respectively). The surface charge of the nanohybrid (−28.8 mV) presents a more negative potential value than MSN-N_3_ (−18 mV), which is consistent with the inclusion of DBCO-CatNCs (−24 mV).

Since CatNCs were chosen as a model of active “soft” nanoparticle, a specific assay was mandatory in order to evaluate the effect of the microfluidic assembly on the activity of the enzyme. Thus, an activity assay was performed to compare the activity of the assembled DBCO-CatNCs, isolated CatNCs, and the free enzyme as a positive control. The enzymatic activity assay confirmed the expected decline in specific activity (U/µg) produced by polymer encapsulation and subsequent functionalization steps (Figure 5C). Free catalase exhibited the highest activity, while DBCO-CatNCs retained 91% of their enzymatic function, indicating that the NHS-mediated coupling and polymerization process preserved catalase structure and function to a significant extent. Upon conjugation to MSN-N_3_, a further drop in specific activity was observed in assembled samples (~74% of retained function). This result may reflect the combined effects of steric hindrance, potential diffusion limitations, or partial inactivation during the click reaction. However, this slight decrease in the activity of the enzyme, besides being expected because of the manipulation of the native enzyme, is worth the decrease since it provides a protective shell to the enzyme against the aggression of the biological environment where this nanoassembly would be used [41].

### 3.5. Colloidal Stability of the Nanosystems

As previously mentioned, preventing nanoparticle aggregation is essential, since exceeding a certain size threshold increases the risk of sedimentation of the suspension. This phenomenon is particularly relevant for MSNs, which tend to form aggregates when no surface modification or coating is present to counteract it. The effect is even more pronounced in aqueous dispersants such as the physiological media, where sedimentation can lead to severe health risks for the patient, including cytotoxicity, embolism, and ischemia [42,43]. Moreover, the potential crosslinking between two complementary Click chemistry-bearing entities could further compromise the colloidal stability of the nanosystem.

**Figure 6:**
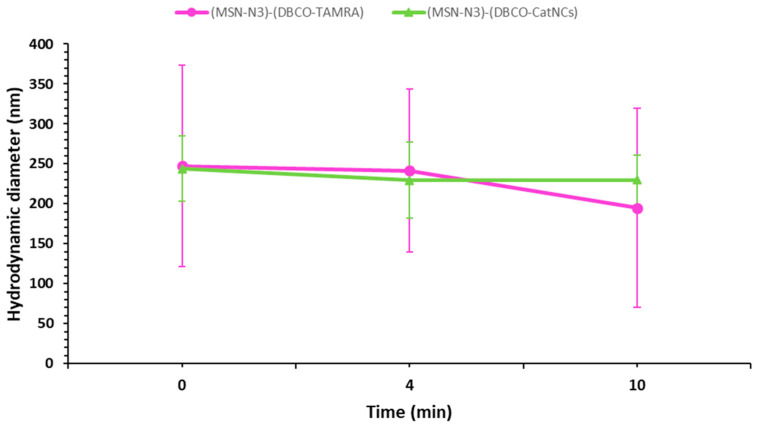
Mean hydrodynamic diameter of the silica and fluorophore conjugate ([MSN-N_3_)-(DBCO-TAMRA)]) and the silica and enzymatic nanocapsule nanoassembly ([MSN-N_3_)-(DBCO-CatNCs)]) over time. Results show that although aggregation is evident in both groups due to an elevated hydrodynamic diameter value, the values maintained a stable colloidal stability well over the residence time of the reagents in the microreactor.

For these reasons, the colloidal stability of both nanoassemblies was evaluated to confirm that they remained in suspension long enough to pass through the microreactor channel. Results showed that although the assemblies exhibited relatively high hydrodynamic diameters, their size remained stable for more than twice the residence time of the microreactor reaction (4.5 min). This indicates reduced and controlled sedimentation of the suspended particles (Figure 6).

### 3.6. Discussion

In previous works, microfluidic platforms have been successfully employed for the surface functionalization or conjugation of nanoparticles under rapid, high-efficiency conditions. For example, Gimondi et al. demonstrated that microfluidic devices allow precise control over nanoparticle migration and surface modification by modulating flow parameters and chip design, thereby improving functionalization homogeneity and reproducibility compared with bulk methods [9]. Likewise, Fabozzi et al. reviewed the state-of-the-art microfluidic strategies for the assembly and functionalization of polymer, lipid, inorganic, and hybrid nanoparticles, emphasizing that continuous-flow microreactors can deliver tunable physical and chemical properties, with minimal batch-to-batch variability and high throughput [44]. These reports align with our findings: although our microreactor does not present a statistically significant increase in functionalization yield over the conventional vial reaction, it achieves equivalent efficiency in only 4.5 min, validating the microfluidic approach as a viable and robust method for rapid nanoparticle conjugation. Moreover, our system offers the added benefits of fine reproducibility, reagent economy, and the prospect of modular integration for sequential syntheses, advantages that are underscored repeatedly in the microfluidic NP literature.

## 4. Conclusions

A low-cost, *homemade* microreactor is proposed in this work as a versatile platform to achieve surface modification of different nanomaterials using click chemistry reactions as covalent bond generation triggers.

The methodology proposed here consisted of the synthesis by traditional methods of model nanoparticles of different chemical nature, in order to test the versatility of the surface modifications to be accomplished within the microfluidic reactor. Specifically, liposomes and mesoporous silica were used as representatives of soft and hard nanoparticles. Once synthesized and a reactive azide -N_3_ group included on their surface, the nanoparticles were characterized in terms of hydrodynamic diameter and surface potential. These values provided a reference to evaluate the occurrence of the subsequent reaction. Afterward, the bare nanoparticles were decorated with a model alkyne bearing an organic model molecule (DBCO-TAMRA) using the microfluidic reactor printed. This chemical modification of the surface with the inclusion of an organic entity was confirmed, and the functionalization yield was estimated, proving that the platform designed is suitable to carry out the chemical modification of the nanoparticle’s surface.

Afterward, a second challenge was faced: the preparation of hybrid nanoassemblies using the microfluidic reactor. This hypothesis was tested using a silica hard nanoparticle and an active polymeric-enzyme containing soft nanoparticle as models. The chemical principle was consistent with the previous approach: the preparation of such a nanohybrid arose from the click reaction of azide and alkine moieties present in each nanoparticle. The success of the nanohybrid assembly was tested by transmission electron microscopy, DLS measurements, and an enzyme activity assay.

The success of the strategy developed relies on the accomplishment of click chemistry reactions, which have proved to be a brilliant tandem together with microfluidics, yielding the desired modified nanoparticles in short times under mild biocompatible conditions. The microfluidic approach envisioned here is also useful not only for the surface modification of different nanoparticles, but also for the assembly of nanostructures, yielding sophisticated multifunctional nanotools.

## Figures and Tables

**Table 1:** DLS and ζ-potential characterization results from the different NP batches. Lip-N_3_: liposomes functionalized with azide groups; MSN-NH_2_ and MSN-N_3_: silica nanoparticles functionalized with amino or azide groups, respectively; CatNCs: polymeric catalase nanocapsules; DBCO-CatNCs: CatNCs functionalized with DBCO; [(Lip-N_3_)-(DBCO-TAMRA)], [(MSN-N_3_)-(DBCO-TAMRA)] and [(MSN-N_3_)-(DBCO-CatNCs)]: nanoassemblies formed via SPAAC reaction between azide-functionalized particles and DBCO-modified components (fluorophore or nanocapsules).

Sample	Hydrodynamic Diameter (nm)	ζ-Potential (mV)
Lip-N_3_	82 ± 6	−5 ± 1
MSN-NH_2_	154 ± 10	18 ± 1
MSN-N_3_	136 ± 3	−18 ± 1
CatNCs	102 ± 47	−21 ± 2
DBCO-CatNCs	50 ± 4	−24 ± 1
[(Lip-N_3_)-(DBCO-TAMRA)]	70 ± 9	−16 ± 5
[(MSN-N_3_)-(DBCO-TAMRA)]	129 ± 15	−7 ± 1
[(MSN-N_3_)-(DBCO-CatNCs)]	267 ± 33	−28.8 ± 0.4

## Data Availability

The original contributions presented in this study are included in the article. Further inquiries can be directed to the corresponding authors.

## Abbreviations

The following abbreviations are used in this manuscript:

NP	Nanoparticle
FDM	Fusion deposition modeling
MCM-41	Mobil Composition of Matter No. 41
MSN	Mesoporous silica nanoparticle
MSN-NH_2_	Aminated mesoporous silica nanoparticles
MSN-N_3_	Azide-functionalized MSNs
Lip-N_3_	Azide-functionalized liposomes
NC	Nanocapsule
CatNC	Catalase NC
SPAAC	Strain-promoted azide-alkyne cycloaddition
TEOS	tetraethyl orthosilicate
APTES	3-aminopropyltriethoxysilane
DIC	N,N′-diisopropylcarbodiimide
NHS	N-hydroxysuccinimide
DBCO	dibenzocyclooctyne
DBCO-TAMRA	DBCO-5-carboxytetramethylrhodamine
CatNC	Catalase nanocapsules
DBCO-CatNC	DBCO-functionalized CatNC
6-AHA	6-azidohexanoic acid
DBCO-NHS	Dibenzocyclooctyne-NHS ester
TMEDA	N,N,N′,N′-tetramethylethylenediamine
AA	Acrylamide
AM	aminoethyl methacrylamide
MBA	N,N′-methylenebisacrylamide
APS	Ammonium persulfate
PEG	polyethylene glycol
PBS	Phosphate-buffered saline
EtOH	Ethanol
DMF	N,N-dimethylformamide
DMSO	Dymtethyl sulfoxide
NH_4_NO_3_	Ammonium nitrate
Na_2_CO_3_	Sodium carbonate
CHCl_3_	Chloroform
NH_4_OH	Ammonium hydroxide
DSPE-N_3_	1,2-distearoyl-sn-glycero-3-phosphoethanolamine-N-[azide(polyethylene glycol)-2000]
DSPC	1,2-distearoyl-sn-glycero-3-phosphocholine
Chol	Cholesterol
DLS	Dynamic light scattering
ELS	Electrophoretic light scattering
MWCO	Molecular weight cut-off
rpm	Revolutions per minute
g	Relative centrifugal force
TEM	Transmission electron microscopy
SEM	Scanning electron microscopy
SEC	Size exclusion chromatography
UV/Vis	Ultraviolet-visible spectroscopy
PLA	Polylactic acid
kDa	Kilodalton
mL	Mililiter
µL	Microliter
µmol	Micromol
mV	Milivolt
µm	Micrometer
U/µg	Specific enzymatic activity (Absorbance units/microgram of material)
kCts.	Kilocounts (Scattering intensity in DLS analysis)
